# The assessment of recalled parental rearing: updated psychometric properties and population-based norms of the FEE German version in a representative sample

**DOI:** 10.3389/fpsyg.2026.1561398

**Published:** 2026-02-05

**Authors:** Katja Petrowski, Vera Clemens, Jörg Michael Fegert, Elmar Braehler, David Hennen, Markus Zenger

**Affiliations:** 1 Department of Medical Psychology and Medical Sociology, University Medical Centre, Johannes Gutenberg University Mainz, Mainz, Germany; 2 Internal Medicine Center, University Hospital Carl Gustav Carus, Dresden, Germany; 3 Department of Child and Adolescent Psychiatry and Psychotherapy, Ulm University Medical Center, Ulm, Germany; 4 Clinic and Polyclinic for Psychosomatic Medicine and Psychotherapy, Rostock University Medical Center, Leipzig, Germany; 5 Department of Applied Human Studies, University of Applied Sciences Magdeburg and Stendal, Stendal, Germany; 6 Integrated Research and Treatment Center, University Hospital Leipzig, Leipzig, Germany

**Keywords:** German, population-based norms, psychometric properties, recalled parental rearing behavior (FEE), representative sample

## Abstract

**Aims:**

Parental rearing behavior has long been recognized as a crucial etiological factor contributing to the vulnerability of psychopathology. Clinical researchers have devoted considerable attention to this subject. In pursuit of this objective, it is vital to have efficient instruments able to assess remembered parental rearing behavior within clinical practice. The aim of this study was to conduct a new psychometric evaluation of the German instrument known as the “Fragebogen zum Erinnerten Elterlichen Erziehungsverhalten (FEE),” designed for the assessment of recalled parental rearing behavior. A recently collected representative dataset was used.

**Methods:**

This questionnaire was psychometrically evaluated in a representative sample of the general population (*N* = 2,373) in Germany which included 50.5% women and 49.5% men with a mean age of *M* = 49.3 (SD = 17.5, range = 14–95).

**Results:**

Fathers were rated higher in rejection and punishment, and mothers were rated higher in emotional warmth with low to medium effect sizes. Men reported higher values of rejection and punishment and lower values of emotional warmth in the father version than women. Living in a city goes along with more rejection and punishment as well as more control and overprotection compared to people from rural areas, but effect sizes were small. Reliability indices were high in the first two scales and acceptable in the scale of control and overprotection. Divergent validity was shown by expected correlations with other health-related variables. The three-dimensional structure of the FEE and the measurement invariance regarding gender and age was confirmed by confirmatory factor analyses. Norm values are presented as percent rank scores.

**Conclusion:**

The FEE represents a dependable and valid tool suitable for use in clinical practice. Our data established a connection between remembered parental rearing behavior, life satisfaction, and interpersonal issues, in accordance with existing literature. We also tackled certain challenges related to the retrospective evaluation of parental rearing behavior.

## 1 Introduction

Since parental rearing behavior has a significant influence on child’s overall psycho-social development (Lamborn et al., 1991; Ong et al., 2018) as well as self-regulation, empathy and health in adulthood (Ding and He, 2022; Seroussi and Yaffe, 2020; Wang et al., 2021) perceived parental rearing practices have been highlighted as a significant etiological factor in a vulnerability model of psychopathology (Perris et al., 1980; Zitzmann et al., 2024). Individuals who reported having supportive, non-rejecting, and non-overinvolved parents displayed higher psychological adjustment, experienced less social alienation, and reported higher life satisfaction (Lamborn et al., 1991; Baker and Hoerger, 2012; Winefield et al., 1989). In the mid-to late-health individuals recalling authoritarian parenting showed worse self-rate health and cognitive functioning compared to authoritative recalled parental rearing (Ding and He, 2022). Even after a severe somatic illness such as cancer, the recalled parental rearing predicts the mental health of these long-term cancer survivors (Ernst et al., 2020).

These effects were found to be moderated by age and gender, as parental rearing strategies changed over time/decades due to cultural shifts and updated norms as well as older individuals tended to idealize their parents’ child-rearing behavior more than younger individuals did (Someya et al., 2000). In addition, gender specific effects could be observed. Male subjects reported experiencing more rejecting parental rearing behavior compared to their female counterparts (Akse et al., 2004; Richter et al., 1992). In male adults especially paternal affection was associated with a better mid- and late-life health. Whereby for female in general, paternal as well as maternal, affection was predictive (Ding and He, 2022). Predicting narcissistic personality structures the paternal overprotectiveness predicts significant positive the male narcissism compared to the maternal warmth significant negative the female narcissism (Green et al., 2020).

In these clinical, retrospective studies, the majority of empirical findings were obtained using questionnaires based on retrospective data, due to high costs of longitudinal studies. When longitudinal data was used the low prevalence of psychological symptoms/disorders lead to lower sample size and less power. For the cross-sectional studies following two questionnaires were used: the first one is the Parental Bonding Instrument (PBI; Parker et al., 1979) and its clinical version, the Measure of Parenting Style (MOPS; Parker et al., 1997). The PBI consists of two dimensions, namely “care” and “control,” while the MOPS adds an additional third dimension of “parental abuse” [retest-reliability = 0.63 to 0.76 (Parker et al., 1979)]. The second questionnaire used is the Egna Minnen Beträffande Uppfostran [EMBU, Own Memories of Child Rearing Experiences (Perris et al., 1980)], which yielded a three-factor structure comprising the dimensions “rejection/punishment,” “emotional warmth,” and “control/overprotection” for both the mother and the father (Arrindell and van der Ende, 1984). The long version of the EMBU displayed an internal reliability of <0.70 (Arrindell and van der Ende, 1984), while the short version showed an internal reliability of >0.72 (Arrindell et al., 1999).

The German 24 – item version of the EMBU, known as the “Fragebogen zum Erinnerten Elterlichen Erziehungsverhalten” (FEE, Recalled Parental Rearing Behavior; Schumacher et al., 2000), has been utilized in various studies exploring perceived parental rearing behavior in siblings and clinical samples, particularly in relation to attachment and relationship characteristics (Albani et al., 2002; Albani et al., 2000; Dick et al., 2005; Kitze et al., 2007; de Roo et al., 2023; Schumacher et al., 1999). Nonetheless, the psychometric properties of this German FEE 24-item version were established years ago in a representative sample. As a result, the objective of this study was to assess the psychometric properties of the 24-item version using a newly collected representative sample. This is necessary since the time/decades changed the parental reading style due to cultural shifts and updated norms. To our knowledge an investigation of the psychometric properties in a recent representative sample is still missing.

The aim of this study was the analysis of psychometric properties with regard to descriptive item characteristics, reliability and aspects of divergent as well as factorial validity of this German FEE short version based on a newly collected representative German sample. It can be hypothesized that this questionnaire may show a similar range of internal reliability indices as published for the different English EMBU versions. In addition, the construct validity of this German FEE short version was evaluated.

Since age and gender showed to be an important modulator of the personality structure and a better mid- and late-life health (Ding and He, 2022; Someya et al., 2000; Akse et al., 2004; Richter et al., 1992; Green et al., 2020), the invariances concerning age and gender should be re-analyzed more in detail based on a representative sample. To the best of our knowledge there is not a recent publication of gender and age invariances based on a representative sample. Concerning the invariance for age and gender, it can be hypothesized that male subjects may state more rejection in parental rearing behavior than female ones. Thereby, it can be hypothesized that older subjects may idealize parental rearing behavior more than younger subjects will.

## 2 Method

### 2.1 Participants and procedures

The present study is based on a representative survey of the German general population in 2020. Data were collected by the Independent Service for Surveys, Methods and Analyses (USUMA, Berlin). The Sampling was conducted using a random selection procedure in three steps. In the first step, the whole country was divided into 258 selection areas, representing non-overlapping inhabited areas in Germany. In a second step, households within each regional sampling area were selected using the random-route-technique. For each area, a starting address and a fixed step interval were predefined. Interviewers had t follow a standardized walking route, listing doorbells or households according to the specified step interval until the required number of addresses were reached. The third step was used to randomly select the household member within each contacted household. For this purpose, the Kish grid method was used as follows. All household members aged 14 years or older were listed on the address sheet according to standardized instructions: men in descending order of age in boxes 1–4 and women in boxes 5–8. Each address sheet contained a pre-assigned random number sequence indicating which box correspond to the selected respondent and the person whose box number appeared first in this sequence was designated as the target person for the interview.

Comparing the present sample with information from the Federal Center for Political Education, it can be assumed fairly representative of the general population concerning age and gender, with a slightly lower proportion of participants aged 70 or older represented in the study sample (Bundeszentrale für Politische Bildung, 2024). Participants were interviewed face to face in their homes by trained interviewers. The first attempt to contact participants was made for 5,676 addresses, of which 5,668 were valid. Out of the initial sample, the study sample consisted of 2,503 men and women (participation rate: 44.1% of valid addresses). After the exclusion of all participants with missing values, the final study sample consisted of 2,373 participants. The percentage of women was 50.5% (*N* = 1,199). Further characteristics of the study sample are given in Table 1.

**Table 1:** Sociodemographic characteristics of the study population.

Sociodemographic characteristics	Total *N* = 2,373	Men *N* = 1,174	Women *N* = 1,199
	*M* (SD)	*M* (SD)	*M* (SD)
Age, years	49.29 (17.49)	48.90 (17.44)	49.66 (17.54)
Age range	14–95	14–93	14–95
Age groups	*N* (%)	*N* (%)	*N* (%)
≤29 years	386 (16.3)	199 (17.0)	187 (15.6)
30–39 years	373 (15.7)	194 (16.5)	179 (14.9)
40–49 years	401 (16.9)	188 (16.0)	213 (17.8)
50–59 years	501 (21.1)	232 (19.8)	269 (22.4)
60–69 years	384 (16.2)	208 (17.7)	176 (14.7)
≥70 years	327 (13.8)	152 (13.0)	175 (14.6)
Missing	1 (<0.1)	1 (<0.1)	
Relationship status			
Married/living together	1,058 (44.6)	557 (47.4)	501 (41.8)
Married/separated	51 (2.1)	22 (1.9)	29 (2.4)
Unmarried	698 (29.4)	403 (34.4)	295 (24.6)
Divorced	350 (14.7)	146 (12.4)	204 (17.0)
Widowed	211 (8.9)	44 (3.7)	167 (13.9)
Missing	5 (0.2)	2 (0.2)	3 (0.3)
Living in partnership			
Yes	1,452 (61.2)	765 (65.2)	687 (57.3)
No	893 (37.6)	397 (33.8)	496 (41.4)
Missing	28 (1.2)	12 (1.0)	16 (1.3)
Education			
≤10 years	1,044 (44.0) 1758 (74.1)	507 (43.2) 848 (72.2)	537 (44.8) 910 (75.9)
>10 years	578 (24.4)	306 (26.1)	272 (22.7)
School student	35 (1.5)	18 (1.5)	17 (1.4)
Missing	2 (0.1)	2 (0.2)	
Employment status			
Education/training	153 (6.4)	74 (6.3)	79 (6.6)
Working	1,407 (59.3)	744 (63.4)	663 (55.3)
Unemployed/working <15 h per week	179 (7.6)	70 (6.0)	109 (9.0)
House wife/man	53 (2.2)	5 (0.4)	48 (4.0)
Retired	579 (24.4)	279 (23.8)	300 (25.0)
Missing	2 (0.1)	2 (0.2)	
Equivalent income in Euro			
<1,250	533 (22.5)	239 (20.4)	294 (24.5)
1,250–<1,500	221 (9.3)	81 (6.9)	140 (11.7)
1,500–<1750	451 (19.0)	235 (20.0)	216 (18.0)
1750–<2000	211 (8.9)	101 (8.6)	110 (9.2)
2000–<2,250	360 (15.2)	192 (16.4)	168 (14.0)
≥2,250	569 (24.0)	316 (26.9)	253 (21.1)
Missing	28 (1.2)	10 (0.9)	18 (1.5)
Residence			
Rural (<20,000 residents)	955 (40.2)	479 (40.8)	476 (39.7)
Town (≥20,000 residents)	1,418 (59.8)	695 (59.2)	723 (60.3)

Household equivalence net income was calculated by dividing net household income by the square root of the number of persons living in the household (https://www.oecd.org/els/soc/OECD-Note-EquivalenceScales.pdf).

The present study was approved by the ethics committee of the University of Leipzig (002/20-ek). Furthermore, it adhered to ICH-GCP-guidelines along with the ICC/ESOMAR International Code of Marketing and Social Research Practice. All the participants were informed about the anonymization of personal data, the study procedures and the data collection. All the participants provided verbal informed consent according to German law, which was documented by the interviewer before starting the survey.

### 2.2 Instruments

#### 2.2.1 Questionnaire of recalled parental rearing behavior (Fragebogen zum Erinnerten Elterlichen Erziehungsverhalten; FEE)

The German version of the FEE (Schumacher et al., 1999) was used, which is conceptually based on the original Swedish EMBU. The FEE is a validated 48-item instrument that covers central aspects of memories of perceived parental rearing behavior, separately for the father and the mother with 24 items each. It comprises the following three factor-analytically derived dimensions with 8 items each: (a) rejection and punishment [e.g., “Have you been punished hard by your father, even for trifles (small offenses)?”], (b) emotional warmth (e.g., “Has your father comforted you when you were sad”) and (c) control and overprotection (e.g., “Do you think that your father’s anxiety that something might happen to you was exaggerated?”). Questions about ones own perceived parental rearing behavior can be answered on a four-point Likert scale in respect to how often the person experienced a certain situation during childhood (1 = no, never, 2 = yes, occasionally, 3 = yes, often, 4 = yes, always).

#### 2.2.2 Brief Symptom Inventory – 18 (BSI-18)

The German version of the BSI-18 (Franke, 2000) was used, which is the translation of the original English version of Derogatis (1993). It is an 18-item self-report symptoms inventory designed as a screening tool to detect psychological distress and psychiatric disorders. It consists of three different subscales with 6 items each: Anxiety (e.g., “Nervousness or shakiness inside”), Depression (e.g., “Feeling no interest in things”) and Somatization (e.g., “Faintness or dizziness”). The items are rated on a 5-point Likert scale ranging from 0 (= not at all) to 4 (= extremely). Subscale scores were computed by the sum of the item scores. Reliability coefficients of the three subscales in terms of McDonalds Omega in the present study are: Anxiety = 0.83, Depression = 0.88, Somatization = 0.82.

#### 2.2.3 Additional single questions

For further validation purposes of the FEE, four additional single questions were used that were expected to be related with the construct of parental rearing behavior. The participants were asked whether he or she (a) was frequently verbally harassed or demeaned, (b) was often hustled or punched, (c) often felt that no one from the family loves him or her, (d) lived together with someone with alcohol problems or drug consumption. Answer options were 0 = no and 1 = yes. These items were expected to correlate positively with the subscale of rejection and punishment, negatively with emotional warmth, and closed to zero with control and overprotection.

### 2.3 Data analyses

Psychometric properties as well as reliability, divergent validity and the potential associations with socioeconomic variables were calculated using IBM SPSS© version 29. Participants with missing values in at least one of the 48 items of the FEE were excluded from the analyses (*N* = 130). For mean comparisons between two subgroups (e.g., men and women) independent *t*-tests were used, for more than two subgroups (age groups) ANOVA was used. In case of dependent variables (assessing the same items in the mother and the father version), the *t*-test for dependent samples was used. To test for relationships between FEE subscales and other health related variables and further single questions, Pearson correlation coefficients were used. The originally postulated three-factorial structure of the FEE was tested using the confirmatory factor analysis (CFA), calculated with AMOS© 27. Models were tested using covariance matrices, and each model was estimated with the maximum likelihood method approach. Model fit was tested on the basis of the following fit indices: the minimum discrepancy divided by its degrees of freedom (CMIN/DF); the comparative-fit-index (CFI); standardized root mean square residual (SRMR); the root mean square error of approximation (RMSEA) and the Normed Fit Index (NFI). For a good model fit, the ratio CMIN/DF should be ≤2 for a good model fit (or at least ≤3 to be acceptable; Schermelleh-Engel et al., 2003); values of NFI and CFI close to 0.95 or higher are indicative of a good or at least acceptable (>0.90) model fit. Furthermore, RMSEA should be ≤0.05 for a good and ≤0.08 for an acceptable model fit. The respective values for SRMR are ≤0.05 and ≤0.10.

Additional analyses were conducted to test the invariance of the model across gender and age using multi-group CFA. After testing the factorial structure in each subgroup, measurement invariance was tested in three steps using first the configural model (no constraints), followed by a metric invariant model (with unstandardized item loadings constrained to be equal across groups), and a scalar invariant model (with unstandardized item loadings and unstandardized item intercepts simultaneously constrained to be equal across groups). Based on the hierarchy of these nested and increasingly restrictive models, the models were then compared to each other. Since the *χ*^2^ statistic has often been criticized for its sensitivity to sample size, we focused mainly on the differences ΔCFI and ΔRMSEA. Values smaller than 0.01 indicate the invariance of the models (Cheung and Rensvold, 2002). To avoid the potential problem of selecting a marker variable that is possibly not invariant, the variance of each latent variable was fixed to 1.0 (and the mean was fixed to 0.0) for scaling purposes (Little et al., 2006).

## 3 Results

### 3.1 Descriptive item and scale analyses

Descriptive item statistics are reported in Table 2.

**Table 2:** Descriptive item characteristics, mean differences and factor loadings.

Item number		*M*	*SD*	*p (d [CI])*	*γ1*	*γ2*	*r_it_*	*λ*
Scale 1: Rejection and Punishment								
FEE Item 1	Father Mother	1.47 1.31	0.69 0.56	<0.001 (0.25 [0.21, 0.29])	1.41 1.87	1.56 3.61	0.74 0.66	0.78 0.70
FEE Item 3	Father Mother	1.53 1.42	0.66 0.59	<0.001 (0.19 [15, 0.23])	1.12 1.27	1.23 1.52	0.69 0.63	0.72 0.67
FEE Item 6	Father Mother	1.30 1.27	0.55 0.53	0.008 (0.05 [0.01, 0.09])	1.82 1.99	2.89 3.76	0.62 0.57	0.64 0.60
FEE Item 8	Father Mother	1.25 1.16	0.58 0.47	<0.001 (0.16 [0.12, 0.20])	2.47 3.31	5.84 11.72	0.80 0.72	0.85 0.80
FEE Item 16	Father Mother	1.27 1.21	0.55 0.49	<0.001 (0.13 [0.09, 0.17])	2.16 2.54	4.86 7.08	0.72 0.67	0.76 0.71
FEE Item 18	Father Mother	1.16 1.10	0.48 0.36	<0.001 (0.14 [0.10, 0.18])	3.32 4.16	11.81 19.74	0.75 0.68	0.80 0.74
FEE Item 20	Father Mother	1.39 1.29	0.63 0.55	<0.001 (0.19 [0.14, 0.23])	1.59 2.04	2.21 4.55	0.77 0.69	0.82 0.76
FEE Item 22	Father Mother	1.28 1.26	0.61 0.57	0.007 (0.06 [0.02, 0.10])	2.37 2.46	5.50 6.21	0.57 0.55	0.61 0.60
Scale 2: Emotional warmth								
FEE Item 2	Father Mother	2.84 3.13	0.91 0.81	<0.001 (−0.43 [−0.39, −0.48])	−0.39 −0.67	−0.64 −0.08	0.77 0.72	0.75 0.73
FEE Item 7	Father Mother	2.45 2.86	0.94 0.86	<0.001 (−0.53 [0.49, −0.58])	−0.01 −0.45	−90. −0.39	0.77 0.73	0.80 0.75
FEE Item 9	Father Mother	2.76 2.98	0.98 0.90	<0.001 (−0.32 [−0.28, −0.36])	−0.32 −0.58	−0.91 −0.44	0.73 0.73	0.76 0.76
FEE Item 12	Father Mother	2.50 2.82	0.94 0.87	<0.001 (−0.47 [−0.42, −0.51])	−0.09 −0.37	−0.87 −0.53	0.81 0.76	0.84 0.79
FEE Item 14	Father Mother	2.48 2.75	0.82 0.75	<0.001 (−0.42 [−0.37, −0.46])	−0.08 −0.25	−0.52 −0.19	0.81 0.77	0.84 0.80
FEE Item 15	Father Mother	2.49 2.94	0.92 0.82	<0.001 (−0.60 [−0.55, −0.63])	−0.02 −0.40	−0.83 −0.42	0.83 0.79	0.86 0.83
FEE Item 17	Father Mother	2.56 2.89	0.88 0.82	<0.001 (−0.51 [−0.46, −0.55])	−0.08 −0.38	−0.69 −0.37	0.83 0.80	0.86 0.83
FEE Item 24	Father Mother	2.16 2.69	0.92 0.90	<0.001 (−0.69 [−0.64, −0.73])	0.27 −0.28	−0.86 −0.65	0.72 0.74	0.75 0.77
Scale 3: Control and overprotection								
FEE Item 4	Father Mother	1.92 1.90	0.90 0.88	0.082 (0.04 [−0.00, 0.08])	0.61 0.60	−0.58 −0.59	0.49 0.51	0.58 0.61
FEE Item 5	Father Mother	1.71 1.87	0.74 0.80	<0.001 (−0.26 [−0.22, −0.30])	0.85 0.57	0.37 −0.34	0.46 0.53	0.55 0.61
FEE Item 10	Father Mother	1.55 1.54	0.71 0.71	0.591 (0.01 [−0.03, 0.05])	1.18 1.22	1.06 1.20	0.44 0.44	0.52 0.51
FEE Item 11	Father Mother	1.89 1.85	0.84 0.84	0.005 (0.06 [0.02, 0.10])	0.58 0.61	−0.50 −0.49	0.51 0.49	0.60 0.60
FEE Item 13	Father Mother	1.31 1.56	0.56 0.74	<0.001 (−0.40 [−0.35, −0.44])	1.80 1.14	3.07 0.59	0.32 0.36	0.38 0.42
FEE Item 19	Father Mother	1.74 1.76	0.83 0.81	0.066 (−0.04 [−0.08, 0.00])	0.97 0.88	0.30 0.19	0.38 0.40	0.46 0.45
FEE Item 21	Father Mother	2.22 2.11	0.83 0.79	0.001 (0.16 [0.12, 0.20])	0.31 0.35	-0.44 −0.29	0.38 0.35	0.48 0.43
FEE Item 23	Father Mother	1.50 1.77	0.67 0.81	<0.001 (−0.43 [−0.39, −0.48])	1.23 0.74	1.16 −0.27	0.45 0.47	0.52 0.54

M = mean; SD = standard deviation; p = *p*-value; d = effect size Cohens *d*; CI = confidence interval of Cohens d; γ_1_ = skewness; γ_2_ = kurtosis; r_it_ = corrected item-total-correlation; λ = standardized factor loading in initial CFA.

Mean differences between the evaluation of experienced behavior of mothers and fathers differed with regard to the scales the items belong to. Concerning items of the scale Rejection and Punishment, fathers had higher scores than mothers, with low effect sizes. Regarding the scale Emotional Warmth, an opposite trend emerged, with medium effect sizes. Further, there is no clear trend in the scale Control and Overprotection.

Concerning the skewness, there is a clear right skewed distribution in the scale Rejection and Punishment, which was to be expected with regard to the construct measured. The skewness of the items of the scale Emotional Warmth is close to zero, indicating a normal distribution, and the skewness values of the scale Control and Overprotection are slightly right skewed. Kurtosis values indicate a clearly peaked distribution of all items of scale 1 and an approximately normal distribution of scale 2 and scale 3 items.

Corrected item-total-correlation vary between 0.55 and 0.83 for all items of Scales 1 and 2. Regarding the items of the third scale, corrected item-total-correlation are lower to some extent.

Concerning descriptive scale characteristics, the same tendencies emerged with regard to means, skewness and kurtosis. Values are shown in Table 3.

**Table 3:** Descriptive scale characteristics and reliability coefficients.

FEE subscale		*M*	SD	*α*	*Ω*	*r*_tt_	*γ*1	*γ*2
Scale 1: Rejection and punishment	Father Mother	10.65 10.02	3.71 3.04	0.91 0.88	0.91 0.88	0.90 0.87	2.20 2.65	5.67 9.73
Scale 2: Emotional warmth	Father Mother	20.24 23.07	6.10 5.48	0.94 0.93	0.94 0.93	0.93 0.92	−0.19 −0.52	−0.67 −0.25
Scale 3: Control and overprotection	Father Mother	13.82 14.36	3.63 3.85	0.73 0.75	0.73 0.75	0.70 0.71	0.56 0.60	0.41 0.27

M = mean; SD = standard deviation; α = Cronbach alpha; *r*_tt_ = split half reliability; *γ*_1_ = skewness; *γ*_2_ = kurtosis.

### 3.2 Reliability and scale intercorrelations

Cronbach alpha coefficients as well as McDonalds omega as indicators of internal consistency and coefficients of split half reliability are shown in Table 3. Internal consistency of scale 1 was high (0.80–0.90) to very high (>0.90) in the father and the mother version, respectively, very high in scale 2 for both versions, and acceptable with values >0.70 in scale 3. Split half coefficients were high in scale 1, very high in scale 2, and acceptable in scale 3.

Scale intercorrelations of the subscales of the FEE are shown in Table 4. The strongest correlations were found between the mother and the father version in the scales of Emotional Warmth and Control and Overprotection, respectively (values >0.70). Nearly zero correlations were found between the scales Emotional Warmth and Control and Overprotection. All other coefficients were of small to medium size and in the expected direction.

**Table 4:** Subscale intercorrelations.

FEE subscale	1	2	3	4	5	6
1 Rejection and punishment –Father		−0.47***	0.35***	0.56***	−0.33***	0.22***
2 Emotional warmth – Father			0.06**	−0.31***	0.74***	0.05*
3 Control and overprotection – Father				0.19***	0.07**	0.75***
4 Rejection and punishment – Mother					−0.48***	0.30***
5 Emotional warmth – Mother						0.07***
6 Control and overprotection – Mother						

*p < 0.05, **p < 0.01, ***p < 0.001.

### 3.3 Divergent validity

As indicators of divergent validity of the FEE subscales scores, Pearson correlation coefficients with other health-related variables as well as the four single questions were calculated. The results are presented in Table 5.

**Table 5:** Pearson correlations between FEE subscale scores and further psychological scales and single items.

FEE subscale	BSI – anxiety	BSI – depression	BSI – somatization	SI1 – harassed or demeaned	SI2 – hustled or punished	SI3 – loved by no one	SI4 –alcohol or drugs
Rejection and punishment – Father	0.29***	0.31***	0.29***	0.53***	0.51***	0.33***	0.33***
Emotional warmth – Father	−0.22***	−0.31***	−0.22***	−0.34***	−0.30***	−0.28***	−0.26***
Control and overprotection – Father	0.17***	0.19***	0.11***	0.19***	0.14***	0.12***	0.10***
Rejection and punishment – Mother	0.31***	0.34***	0.29***	0.42***	0.39***	0.28***	0.27***
Emotional warmth – Mother	−0.23***	−0.30***	−0.23***	−0.31***	−0.25***	−0.29***	−0.21***
Control and overprotection – Mother	0.17***	0.18***	0.11***	0.14***	0.11***	0.09***	0.11***

SI = single items; ***p < 0.001.

As shown, all correlation coefficients are in the expected direction, total values are of small to medium size and statistically significant. Therefore, the results indicate the divergent validity of the FEE.

### 3.4 Factorial validity of the FEE

The factorial structure of the FEE – consisting of three correlated latent factors with eight items each was tested using CFA, separately for the father and mother version. Fit indices are given in Table 6.

**Table 6:** Summary of fit indices of different factor models.

Model	*χ*^2^ (df)	CMIN/DF	CFI	SRMR	RMSEA (CI)	NFI
Three-factor model – father	2891.222 (249)	11.611	0.914	0.078	0.067 (0.065–0.069)	0.907
Modificated three-factor model – father	2051.871 (247)	8.307	0.941	0.072	0.056 (0.053–0.058)	0.934
Three-factor model – mother	2364.037 (249)	9.494	0.918	0.068	0.060 (0.058–0.062)	0.909
Modificated three-factor model – mother	1667.425 (247)	6.751	0.945	0.064	0.049 (0.047–0.051)	0.936

df = degrees of freedom; CMIN/DF = minimum discrepancy, divided by its degrees of freedom; CFI = comparative-fit-index; SRMR = standardized root mean square residual; RMSEA (CI) = root mean square error of approximation (confidence interval); NFI = Normed Fit Index.

The model showed a good or at least an acceptable model fit regarding all but one fit indices. Modification indices indicated a substantial improvement of model fit when allowing several error terms to correlate. This step is allowed only when also stating a reason with regard to the content of the items. Therefore, the error terms of two item pairs were allowed to correlate (item 1 and 3 as well as item 4 and 11), because the item content is very similar and, thus, the error terms might share a common variance that is not covered by the latent factor. This procedure resulted in an improvement of the model fit as shown in Table 6 for both the mother and the father version.

Additionally, measurement invariance across gender and different age groups was tested using multi-group CFA. The results are shown in Table 7 for the father version and Table 8 for the mother version of the FEE. As the indices of *ΔCFI* and *ΔRMSEA* indicate (<0.01), this model can be assumed to be scalar invariant across males and females as well as across six age groups. The Δ*χ*^2^ statistic indicated significant differences in all cases of the invariance tests, but due to its sensitiveness to sample size we focused on differences in values RMSEA and CFI (Schermelleh-Engel et al., 2003).

**Table 7:** Tests for invariance across gender and age groups for FEE – father.

Invariance test	*N*	*χ*^2^ (df)	Δ *χ*^2^	Δ *p*	CMIN/DF	CFI	Δ CFI	RMSEA	Δ RMSEA
Gender									
Men	1,174	1136.876 (247)			4.603	0.940		0.055	
Women	1,199	1226.080 (247)			4.964	0.938		0.058	
Multigroup analysis									
Configural model		2362.956 (494)			4.783	0.939		0.040	
Metric model		2430.622 (518)	67.667	<0.001	4.692	0.938	0.001	0.039	0.001
Scalar model		2678.043 (542)	247.420	<0.001	4.941	0.930	0.008	0.041	0.002
Age									
≤29 years	386	669.529 (247)			2.711	0.921		0.067	
30–39 years	373	633.379 (247)			2.564	0.919		0.065	
40–49 years	401	529.492 (247)			2.144	0.945		0.053	
50–59 years	501	640.888 (247)			2.595	0.938		0.056	
60–69 years	384	559.563 (247)			2.265	0.936		0.057	
≥70 years	327	659.692 (247)			2.671	0.903		0.072	
Multigroup analysis									
Configural model		3692.709 (1482)			2.492	0.928		0.025	
Metric model		3903.246 (1602)	210.537	<0.001	2.436	0.925	0.003	0.025	0.000
Scalar model		4190.358 (1707)	287.111	<0.001	2.433	0.920	0.005	0.025	0.000

df = degrees of freedom; CMIN/DF = minimum discrepancy, divided by its degrees of freedom; CFI = Comparative-Fit Index; RMSEA = root mean square error of approximation.

**Table 8:** Tests for invariance across gender and age groups for FEE – mother.

Invariance test	*N*	*χ*^2^ (df)	Δ*χ*^2^	Δ *p*	CMIN/DF	CFI	Δ CFI	RMSEA	Δ RMSEA
Gender									
Men	1,174	965.612 (247)			3.909	0.939		0.050	
Women	1,199	1042.416 (247)			4.220	0.943		0.052	
Multigroup analysis									
Configural model		2008.028 (494)			4.065	0.941		0.036	
Metric model		2071.259 (518)	63.231	<0.001	3.994	0.940	0.001	0.036	0.000
Scalar model		2130.270 (542)	59.011	<0.001	3.926	0.939	0.001	0.035	0.001
Age									
≤29 years	386	591.702 (247)			2.396	0.922		0.060	
30–39 years	373	602.523 (247)			2.439	0.914		0.062	
40–49 years	401	508.239 (247)			2.058	0.940		0.051	
50–59 years	501	602.785 (247)			2.440	0.931		0.054	
60–69 years	384	537.745 (247)			2.177	0.936		0.055	
≥70 years	327	559.175 (247)			2.264	0.911		0.062	
Multigroup analysis									
Configural model		3402.292 (1482)			2.296	0.927		0.023	
Metric model		3616.679 (1602)	174.607	<0.001	2.254	0.923	0.004	0.023	0.000
Scalar model		3857.508 (1707)	280.609	<0.001	2.260	0.917	0.006	0.023	0.000

df = degrees of freedom; CMIN/DF = minimum discrepancy, divided by its degrees of freedom; CFI = Comparative-Fit Index; RMSEA = root mean square error of approximation.

### 3.5 Mean differences with regard to sociodemographic variables

Several *t*-tests (for gender and residence) and an ANOVA (for six age groups, see Tables 9, 10) were conducted to test for mean differences in the subscales of the FEE.

**Table 9:** Mean differences with regard to sociodemographic variables: gender and residence.

Invariance test	Gender			Residence		
	Men	Women	*p* (*d[CI]*)	Rural	Town	*p* (*d[CI]*)
Rejection and punishment Father [mean (SD)]	10.99 (3.84)	10.33 (3.56)	<0.001 (0.18 [0.10, 0.26])	10.46 (3.58)	10.79 (3.80)	0.034 (−0.09 [−0.17, −0.01])
Rejection and punishment Mother [mean (SD)]	9.99 (2.86)	10.04 (3.21)	0.682 (−0.02 [−0.10,0.06])	9.82 (2.78)	10.14 (3.20)	0.010 (−0.11 [−0.19, −0.02])
Emotional warmth Father [mean (SD)]	19.61 (6.00)	20.86 (6.14)	<0.001 (−0.21 [−0.29, −0.13])	20.32 (5.97)	20.18 (6.19)	0.583 (0.02 [−0.06, 0.11])
Emotional warmth Mother [mean (SD)]	22.87 (5.31)	23.26 (5.65)	0.087 (−0.07 [−0.15, 0.01])	23.19 (5.33)	22.99 (5.58)	0.359 (0.04 [–0.04, 0.12])
Control and overprotection Father [mean (SD)]	13.82 (3.53)	13.82 (3.72)	0.994 (0.00 [−0.08, 0.08])	13.55 (3.54)	14.01 (3.67)	0.003 (−0.13 [−0.21, −0.04])
Control and overprotection Mother [mean (SD)]	14.36 (3.82)	14.36 (3.88)	0.957 (<−0.01 [−0.08, 0.08])	14.02 (3.81)	14.59 (3.85)	<0.001 (−0.15 [−0.23, −0.07])

d = effect size Cohens d; CI = confidence interval of Cohens *d*.

**Table 10:** Mean differences with regard to sociodemographic variables: age.

FEE subscale	Age groups							
	1	2	3	4	5	6	p (*η [CI]*)	s.d.
Rejection and punishment Father [mean (SD)]	10.13 (3.55)	10.34 (3.27)	10.39 (3.50)	10.70 (3.85)	11.07 (3.87)	11.40 (4.08)	<0.001 (0.01 [0.00, 0.02])	6–1/2/3; 5–1
Rejection and punishment Mother [mean (SD)]	9.85 (3.18)	10.03 (3.06)	9.81 (2.74)	9.94 (2.81)	10.27 (3.41)	10.27 (3.09)	0.145 (<0.01 [0.00, 0.01])	–
Emotional warmth Father [mean (SD)]	21.24 (5.52)	20.82 (6.12)	21.23 (6.01)	20.13 (6.11)	18.86 (5.94)	18.95 (5.45)	<0.001 (0.03 [0.01, 0.04])	6–1/2/3; 5–1/2/3/4
Emotional warmth Mother [mean (SD)]	24.52 (5.52)	23.53 (5.49)	23.39 (5.23)	22.68 (5.32)	21.89 (5.54)	22.41 (5.12)	<0.001 (0.02 [0.01, 0.04])	6–1;5–1/2/3; 4–1
Control and overprotection Father [mean (SD)]	13.97 (3.93)	13.53 (3.70)	14.17 (3.51)	13.73 (3.59)	13.76 (3.57)	13.79 (3.43)	0.189 (<0.01 [0.00, 0.01])	–
Control and overprotection Mother [mean (SD)]	14.83 (4.02)	14.28 (3.86)	14.56 (3.70)	14.28 (3.92)	14.19 (3.90)	14.01 (3.58)	0.060 (<0.01 [0.00, 0.01])	–

η = effect size Eta squared; CI = confidence interval of Eta squared; s.d. = significant differences between the subgroups.

Mean differences according to several sociodemographic variables were relatively small in magnitude, but some general trends emerged. Men reported higher values of rejection and punishment as well as lower values of emotional warmth in the father version than women. Regarding the location of residence, participants living in cities reported more rejection and punishment as well as more control and overprotection in the father and the mother version compared to those living in rural areas, but effect sizes were very small. With regard to the different age groups, there seems to be a linear trend. The older the participants, the higher the values in the subscale of rejection and punishment in the father version, but no differences in the mother version. The opposite is true for the subscales of emotional warmth in both versions, but this tendency is not so strict. No significant differences were found in the subscales of control and overprotection.

### 3.6 Normative values

Percent rank scores for all three FEE scales are shown separately for the mother and the father version in Table 11.

**Table 11:** Normative percent rank scores for the FEE mean scores.

(Rounded) percent rank	FEE – Rejection and punishment Father	FEE – Rejection and punishment Mother	FEE – Emotional warmth Father	FEE – Emotional warmth Mother	FEE – Control and overprotection Father	FEE – Control and overprotection Mother
10	–	–	11	15	9	9
20	–	–	14	18	10	10
30	–	–	16	20	11	11
40	8	8	18	22	12	12
50	9	–	20	23	13	13
60	10	9	22	24	14	15
70	11	10	23	26	15	16
80	12	11	25	27	16	17
90	15	13	28	29	18	19

## 4 Discussion

The primary objective of this study was to assess psychometric properties with regard to descriptive item characteristics, reliability and aspects of divergent as well as factorial validity of the FEE questionnaire in a recently collected representative data set. Additionally, we explored how sociodemographic factors might be associated with individuals’ perceptions of parental rearing behavior. This is necessary since the time/decades changed the parental reading style due to cultural shifts and updated norms. To our knowledge an investigation of the psychometric properties in a recent representative sample is still missing.

In this study, item characteristics showed that the recalled behavior of fathers scored higher in terms of rejection and punishment and mothers scored higher in emotional warmth. Reliability coefficients could be shown to be high or at least acceptable, taking the shortness of the scales into account. Divergent validity was shown by correlations with other health-related constructs that revealed significant relationships in the expected directions and with an expected amount. Corrected item-total-correlation and standardized factor loadings of all items are substantially high, with one exception. Regarding the factor of Control and Overprotection, both indices are considerably lower (especially for items 13, 19 and 21), which might reflect the heterogeneity of this sub-construct. This explanation remains to some extent speculative, as it does not rule out alternative explanations (e.g., ambiguous item wording or misalignment between items and cultural context), which cannot be tested in the current data set. This limitation weakens the interpretability of the Control and Overprotection subscale, as its assessment is less precise compared to the other subscales. These findings from the confirmatory factor analyses (CFAs) demonstrated an overall strong alignment with the three-factor model for both paternal and maternal items under the condition that two pairs of item errors were allowed to correlate, which might hamper the factorial validity to a small extent. This provided confirmation for the previously assumed item allocation, which was adapted from the German translated version of the EMBU [FEE, as referenced in Lamborn et al. (1991) and Perris et al. (1980)]. Consequently, individuals’ recollections of their mother’s and father’s rearing behavior could be characterized across three dimensions: rejection and punishment, emotional warmth, and control and overprotection. These three distinct scales for each parent effectively captured the recalled parental rearing behavior.

Since age and gender showed to be an important modulator for recalled parental rearing (Ding and He, 2022; Someya et al., 2000; Akse et al., 2004; Richter et al., 1992; Green et al., 2020), the invariances concerning age and gender should be re-analyzed based on a representative sample. To the best of our knowledge there is not a recent publication of gender and age invariances based on a representative sample.

The stability of these three dimensions remains consistent across genders and age groups, suggesting that the instrument encompasses the same factors when applied to individuals of either gender between the ages of 14 and 95. Therefore, it should be feasible to rank individuals, or more specifically, their memories, along these dimensions. By establishing additionally population-based norms, future studies can provide essential information for individual assessments, enabling individuals to be compared to their respective peer group.

While the existing literature on the EMBU has addressed the influence of gender (Halverson, 1988; Kiviniemi et al., 2016) and age (Perris et al., 1980; Ostgård-Ybrandt and Armelius, 2004; Schneewind et al., 1999) only sparsely or in a non-representative sample, our current representative data has revealed some statistically significant but relatively minor effects of these factors on individuals’ recollections of parental rearing behavior. These effects are similar to previous research results conducted by the authors of the FEE questionnaire (Lamborn et al., 1991).

Our findings indicate that older participants tend to recall their fathers – but not their mothers – as more rejecting. Furthermore, younger participants reported higher values than older ones in the subscale of emotional warmth in both, the mother and the father version of the questionnaire. This suggests that older individuals do not idealize their parental rearing behavior more than younger ones (Perris et al., 1980; Schneewind et al., 1999; Chopik et al., 2023). Additionally, men reported higher rejection values and lower values in emotional warmth in the father version of the FEE compared to women. Regarding the location of residence of the participants, people living in a city reported slightly higher values of rejection and punishment as well as control and overprotection in both, the mother and the father version of the FEE. All effect sizes were relatively small with one exception. Comparing the mother and the father version in the subscale of emotional warmth, medium effects sizes emerged, indicating a stronger practical relevance.

The observed age-related differences might be attributed to historical shifts in parenting attitudes and child-rearing practices within the studied German population. These changes may represent a transition from earlier values, such as discipline and order, prevalent among the older generation, to more child-centered approaches in the post-modern information society, characterized by diversity and abundant information on “good” parenting. To understand these variations fully, it is crucial to consider the specific historical context of each cohort. For instance, individuals who are now in their 80s and older grew up during and shortly after World War II or the following depression, the rebuilding of Germany, experiencing hardship, absent or disabled fathers, malnutrition, and overwhelmed mothers. In contrast, the next generation, born and raised in the 1960s, witnessed rapid economic growth during the German “economic miracle” and lived in an increasingly liberal society following the student uprisings of 1968 across Europe. These societal events most likely influenced the values underpinning child -rearing and the conditions surrounding it. However, it is important to note that further research is needed to determine how these changes affected children from different socio-economic backgrounds.

Concerning gender-related differences, according to their recollections, female participants reported receiving more emotional warmth from their fathers compared to their male counterparts. In contrast, male participants recalled their fathers as being stricter and more rejecting. These differences could potentially be explained by fathers feeling compelled to conform to stereotypical roles when raising their sons, emphasizing attributes like strength and stoicism. These sociodemographic tendencies have practical relevance and should be taken into account when the FEE is used in large-scale preventive studies. Therefore, normative values in a recently collected representative data sample and cutoff points for the FEE have practical relevance in order to facilitate its implementation in the field of prevention. However, it is important to acknowledge that these considerations are limited by the fact that only one cohort from one country was examined, leaving room for speculation regarding the variability and cultural specificity of child-rearing practices elsewhere (Julian et al., 1994; Mousavi et al., 2016).

This study’s notable strengths lie in its substantial and representative sample size as well as its rigorous statistical approach. However, it is important to acknowledge as a limitation that a large sample size can sometimes result in the detection of statistically significant but practically small correlation coefficients, as underscored by the modest effect sizes observed in this study. In addition, retrospectively evaluating recalled parental rearing behavior presents a unique challenge when assessing the actual upbringing experienced during childhood or its subjective representation (Brewin et al., 1993; Gerlsma et al., 1994; Gerlsma, 2000). This subjective representation can be influenced by one’s present mood, errors in autobiographical memory (both conscious and unconscious distortions), false memories, or individualized reconstructions of their personal history. However, the existing body of literature has not yielded consistent or definitive findings regarding the mood-related recall of relevant personal experiences (Julian et al., 1994; Brewin et al., 1993; Gerlsma et al., 1994; Gillham et al., 2007; Matt et al., 1992; Parrott and Sabini, 1990), nor has it provided conclusive evidence on the reliability of retrospective reports on parental rearing behavior (Halverson, 1988; Morsbach and Prinz, 2006). Therefore, it is advisable for future research to consider longitudinal studies that involve independent observers from outside the family to establish the validity of parental rearing practices (Kiviniemi et al., 2016; Nivison et al., 2021). Unfortunately, in clinical practice, assessing the child-rearing experiences of patients can only be carried out retrospectively after the onset of their disorder. Nevertheless, the information obtained at that point can still be valuable to the therapeutic process.

In summary, the present data demonstrate that the 24-item version of the EMBU and its German counterpart, the FEE, the established factorial structure remains intact. This underscores the instrument’s reliability in retrospectively assessing individuals’ subjective perceptions of parental rearing behavior. Therefore, it permits the concurrent assessment of other potential risk factors. Furthermore, this information holds significance not only for research related to psychological disorders but also for non-clinical applications, including prevention projects and counseling settings. By identifying such risk factors through early screening, there is an opportunity to support parents in adopting a more positive parenting style. Subsequently, intervention programs can be tailored more precisely to the population in need, potentially preventing the chronicity of illnesses and the associated costly treatments. The tailored intervention programs should pick up the need-supportive behaviors of the different individuals in a family system (i.e., autonomy, competence and relatedness behaviors) (Ahmadi et al., 2023). Ahmadi et al. (2023) provided a classification system of need-supportive techniques, which can help to tailor an individualized intervention program for this family system.

## Data Availability

The raw data supporting the conclusions of this article will be made available by the authors, without undue reservation.
